# First Case of Typhoid Fever due to Extensively Drug-resistant *Salmonella enterica* serovar Typhi in Italy

**DOI:** 10.3390/pathogens9020151

**Published:** 2020-02-24

**Authors:** Michela Procaccianti, Alice Motta, Stefano Giordani, Sara Riscassi, Battista Guidi, Monica Ruffini, Valentina Maffini, Susanna Esposito, Icilio Dodi

**Affiliations:** 1 Pietro Barilla Children’s Hospital, Department of Medicine and Surgery, University of Parma, 43126 Parma, Italy; michela.procaccianti@outlook.it (M.P.); riscassi.sara@gmail.com (S.R.); ruffiniM@ao.pr.it (M.R.); vamaffini@ao.pr.it (V.M.); idodi@ao.pr.it (I.D.); 2 Paediatric Unit, AUSL of Modena, 41026 Pavullo nel Frignano (MO), Italy; a.motta@ausl.mo.it (A.M.); b.guidi@ausl.mo.it (B.G.); 3 Infectious Diseases Unit, AUSL of Modena, 41026 Pavullo nel Frignano (MO), Italy; s.giordani@ausl.mo.it

**Keywords:** blaCTX-M-15 extended-spectrum β-lactamase gene, carbapenem, XDR *Salmonella enterica* serovar Typhi, *Salmonella* Typhi, typhoid fever

## Abstract

Typhoid fever is a potentially severe and occasionally life-threatening bacteraemic illness caused by *Salmonella enterica* serovar Typhi (*S.* Typhi). In Pakistan, an outbreak of extensively drug-resistant (XDR) *S.* Typhi cases began in November 2016. We report on a five-year-old boy who contracted enteric fever while travelling in Pakistan and was diagnosed after returning to Italy in September 2019. Blood culture isolated *Salmonella enterica* serovar Typhi that was XDR to all first-line antibiotics, including ceftriaxone and fluoroquinolones. Empiric therapy was switched to meropenem, and the patient recovered completely. Whole-genome sequencing showed that this isolate was of haplotype H58. The XDR *S.* Typhi clone encoded a chromosomally located resistance region and harbored a plasmid encoding additional resistance elements, including the blaCTX-M-15 extended-spectrum β-lactamase and the qnrS fluoroquinolone resistance gene. This is the first case of typhoid fever due to XDR *S.* Typhi detected in Italy and one of the first paediatric cases reported outside Pakistan, highlighting the need to be vigilant for future cases. While new vaccines against typhoid are in development, clinicians should consider adapting their empiric approach for patients returning from regions at risk of XDR *S.* Typhi outbreak with typhoid symptoms.

## 1. Introduction

Typhoid fever is a potentially severe and occasionally life-threatening bacteraemic illness caused by *Salmonella enterica* serovar Typhi (*S.* Typhi) [1]. It is endemic in many developing countries where water and food may be unsafe and sanitation is poor and where several million cases occur every year, causing more than 200,000 deaths [1]. Fortunately, in industrialized countries, typhoid fever has become relatively rare. In the USA during 2008–2015, only approximately 350 culture-confirmed cases of typhoid fever were reported to the Centers for Disease Control and Prevention each year, with a rate lower than 0.5 cases per 100,000 population [2]. As this rarity was associated with persistent sensitivity of most *S.* Typhi to antibiotics such as third-generation cephalosporins [3], which were considered to be the first choice for infection treatment since the end of the last century, interest in typhoid fever in industrialized countries has gradually decreased. 

However, in recent years, two problems have raised new interest in the disease: evidence that a sudden increase in the number of typhoid fever diagnoses had occurred in some, if not all, industrialized countries and the demonstration that most of the unexpected cases were due to a *S.* Typhi strain with extensive drug resistance. An increase in typhoid fever cases was evidenced in Australia, Canada, Denmark, Taiwan, Ireland, the United Kingdom and the United States [4,5,6]. In most of these cases, diagnosis was made in subjects with a history of travel from Pakistan, where an outbreak of extensively drug resistant (XDR) *S.* Typhi cases began in November 2016 [7]. This led to the hypothesis that most of these diseases were an extension of the Pakistan epidemic. Bacterial genomic sequencing confirmed the identity between strains and showed that the XDR *S.* Typhi clone harboured a plasmid encoding resistance elements (blaCTX-M-15 extended-spectrum β-lactamase) and carrying the qnrS fluoroquinolone resistance gene [8]. 

As XDR *S.* Typhi can cause severe clinical problems because it is sensitive only to azithromycin and carbapenems, diffusion of this pathogen must be monitored, particularly the emergence of cases in geographic areas previously not impacted by this microbiological problem. This paper reports the first case of XDR *S.* Typhi infection reported in Italy and one of the first cases reported in the paediatric age outside Pakistan. 

## 2. Methods and Results

On September 21, 2019, a previously healthy five-year-old boy was taken to a hospital in Emilia-Romagna Region (Pavullo Hospital, Modena), Italy, with a four-day history of high-grade fever (40°C), vomiting and general abdominal pain. He had a cough but no difficulty breathing. He had recently travelled to Pakistan for a one-year stay and returned to Italy two weeks before admission to the hospital. He had no underlying condition that could explain the symptoms observed or that can render him more susceptible to typhoid fever. In the emergency room, examination revealed an acutely ill child who was febrile at 39.4°C, with a normal heart rate and respiratory rate. A skin examination did not reveal any rash, petechiae, or bruising. A chest and cardiovascular examination revealed no abnormalities. His abdomen was soft with no organomegaly. There was no clinical ascites, and his bowel sound was present. He was alert and oriented, with normal neurological examination. 

Table 1 summarizes laboratory exams at admission, during hospitalization and during follow-up. The initial investigation showed leukopenia, thrombocytopenia and elevated C-reactive protein (CRP). The white cell count was 5300/µL (82.7% neutrophils), reached a nadir of 2700/µL on day five of admission (87.3% neutrophils) and gradually improved to 5000/µL by day seven (81.6% neutrophils) and reached normal values by day 21 (7300/µL, 69.9% neutrophils). The nadir platelet count was 101,000/µL on day three of admission, and the platelet count gradually improved to 144,000/µL. Renal function was normal, while liver function tests showed mild transaminitis with normal bilirubin concentrations.

The patient was hospitalized; blood culture and serological tests were performed, and he started intravenous hydration. Because of persistent daily fever and the appearance of a maculopapular rash on the abdomen and lower chest the day after admission, he was diagnosed with presumed enteric fever, and empirically intravenous ceftriaxone was started. 

*S. enterica* serovar Typhi was isolated from blood culture using routine laboratory methods. Automated antimicrobial susceptibility testing of the isolate (Thermo Scientific Sensititre ARIS HiQ AST System, Waltham, MA USA) identified resistance to all first-line antibiotics, including ceftriaxone (Table 2). When these results became available, empiric ceftriaxone was switched to intravenous meropenem 100 mg/kg/day on day five. The child continued to have daily high-grade fever for three days during meropenem treatment, and then he was afebrile and showed a complete recovery.

Whole-genome sequencing showed that this isolate was of haplotype H58. The XDR *S.* Typhi clone encoded a chromosomally located resistance region and harboured a plasmid encoding additional resistance elements, including the blaCTX-M-15 extended-spectrum β-lactamase and the qnrS fluoroquinolone resistance gene. Using the PlasmidFinder tool, we identified two plasmids: the IncY plasmid, which exhibited 100% sequence identity to the plasmid found in the Pakistan outbreak, and the IncQ1 plasmid. The strain was identical to those involved in the outbreak in Pakistan [8] and no mutation was detected.

## 3. Discussion

The case described here is the first case of typhoid fever due to XDR *S.* Typhi detected in Italy and one of the first paediatric cases reported outside of Pakistan. As previously described in North America, Australia and some European countries [8,9,10], globalization is one of the main causes of the diffusion of microbial resistance to currently used antibiotics and can cause relevant treatment problems for diseases for which a previous therapeutic approach did not require careful handling [4,5,6]. 

Most cases of typhoid fever due to XDR *S.* Typhi infection have been directly imported from Pakistan, where epidemics have begun. However, as typhoid fever can be easily transmitted from human to human by the faecal-oral route, further cases involving the resident population can occur and an epidemic can develop. This means that after a first case of typhoid fever due to an XDR *S.* Typhi strain is diagnosed in a country, all the other cases of the disease in the same country must be adequately investigated and treated in order to address this new microbiological problem. 

The development of *S.* Typhi resistance to antibiotics is an ongoing challenge. This challenge began with evidence of resistance to chloramphenicol, ampicillin, and trimethoprim-sulfamethoxazole, the drugs initially considered the antibiotics of choice to treat *S*. Typhi infections [1]. Drug-resistant *S.* Typhi emerged in the late 1970s to early 1980s and led to the use of fluoroquinolones as a major treatment option for *S.* Typhi cases [1]. However, studies have identified *S.* Typhi isolates resistant to fluoroquinolones, with the first case being reported in 1992 [3,6]. After that, treatment of typhoid fever was based on third-generation cephalosporins, ceftriaxone for parenteral therapy and cefixime for oral therapy [3,6]. Resistance of *S.* Typhi to ceftriaxone remained rare until 2016, when a large outbreak of ceftriaxone-resistant cases was reported in Pakistan [7]. This XDR strain is the cause of the present epidemic and can be treated only with carbapenems and azithromycin. Our patient showed sensitivity to carbapenems, piperacillin/tazobactam and aminoglycosides, whereas azithromycin and other macrolides were not tested. However, it is highly likely that in the future, further therapeutic problems for typhoid fever therapy will emerge. Recent reports indicate the presence of *S. Typhi* strains resistant to azithromycin [11], leading to further limitations of antimicrobial therapy and the need for continuous monitoring of *S.* Typhi sensitivity.

Over the past two decades, the dominant haplotype of *S.* Typhi called H58 has been spreading globally. In H58, as with other *S.* Typhi clades, antimicrobial resistance genes are generally associated with an IncHI1 plasmid. This plasmid harbours a composite transposon that can carry multiple resistance genes, including blaTEM-1 (ampicillin resistance), dfrA7, sul1, sul2 (trimethoprim-sulfamethoxazole resistance), catA1 (chloramphenicol resistance), and strAB (streptomycin resistance) genes [2,3,4,5,6]. This composite transposon has also been integrated into the chromosome in some H58 *S.* Typhi lineages. Reduced susceptibility to fluoroquinolones is associated with chromosomal mutations and acquisition of antimicrobial resistance genes. The acquisition of plasmid-mediated quinolone resistance genes can also contribute to fluoroquinolone resistance. Ceftriaxone resistance was associated with the acquisition of an extended-spectrum β-lactamase gene [6]. The XDR *S.* Typhi clones, defined as strains resistant to first-line drugs, fluoroquinolones, and third-generation cephalosporins, encode a chromosomally located resistance region, harbour a plasmid encoding additional resistance elements, including the blaCTX-M-15 extended-spectrum β-lactamase, and carry the qnrS fluoroquinolone resistance gene [11]. Whole genome sequencing confirmed that the child in this report was infected with the XDR outbreak strain. If an XDR strain is encountered, current empiric antimicrobial choices will result in treatment failure, and carbapenems or azithromycin are the appropriate therapy. 

## 4. Conclusions

This first case of typhoid fever due to XDR *S.* Typhi detected in Italy is one of the first paediatric cases reported outside of Pakistan and highlights the need to be vigilant for future cases. While new vaccines against typhoid are in development, clinicians should consider adapting their empiric approach for patients returning from regions at risk of XDR *S.* Typhi outbreak with typhoid symptoms.

## Figures and Tables

**Table 1:** Laboratory exams at admission, during hospitalization and in follow-up of a five-year-old child with extensively drug resistant (XDR) S. Typhi.

	Admission	Day 2	Day 5	Day 7	Day 21
Clinical findings					
Fever, max AT in °C	39.4	40.1	39.7	37.7	36.4
Other signs symptoms	Cough	Maculo-papular rash on the abdomen and lower chest	Maculo-papular rash on the abdomen and lower chest	None	None
Antibiotic therapy	None	Ceftriaxone	Meropenem	Meropenem	None
Laboratory exams					
WBC, cells/µL	5,300	3,500	2,700	5,000	7,300
Hb, g/dL	10.3	9.7	10.1	11.5	12.8
Platelets, cells/µL	138,000	106,000	101,000	144,000	179,000
CRP, mg/L	163	190	138	49	4
AST, U/L	188	182	179	144	39
ALT, U/L	67	55	50	67	31
Blood culture	Positive for XDR S. enterica serovar Typhi				Negative

ALT, alanine transaminase; AST, aspartate aminotransferase; CRP, C-reactive protein; Hb, haemoglobin; WBC, white blood cells.

**Table 2:** Antimicrobial susceptibility testing of the *S. enterica* serovar Typhi isolate detected in blood culture interpreted by Clinical and Laboratory Standards Institute (CLSI).

Antibiotics	Susceptibility	MIC, µg/mL
Amoxicillin/clavulanic acid	R	≥32
Piperacillin/tazobactam	S	Sensitive
Cefoxitin	-	≤4
Cefotaxime/Ceftriaxone	R	≥64
Ceftazidime	R	≥64
Cefepime	R	16
Ertapenem	S	Sensitive
Meropenem	S	Sensitive
Ciprofloxacin	R	≥4
Co-trimoxazole	R	≥320
Fosfomycin	S	Sensitive
Tigecycline	-	≤0.5
Gentamicin	S	Sensitive
Amikacin	S	Sensitive

MIC, minimum inhibitory concentration; S, susceptible; R, resistant.
